# Huprines as a new family of dual acting trypanocidal–antiplasmodial agents

**DOI:** 10.1016/j.bmc.2011.01.028

**Published:** 2011-03-01

**Authors:** Julien Defaux, Marta Sala, Xavier Formosa, Carles Galdeano, Martin C. Taylor, Waleed A.A. Alobaid, John M. Kelly, Colin W. Wright, Pelayo Camps, Diego Muñoz-Torrero

**Affiliations:** a Laboratori de Química Farmacèutica (Unitat Associada al CSIC), Facultat de Farmàcia, and Institut de Biomedicina (IBUB), Universitat de Barcelona, Av. Diagonal, 643, 08028 Barcelona, Spain; b London School of Hygiene and Tropical Medicine, Department of Infectious and Tropical Diseases, Keppel Street, London WC1E 7HT, United Kingdom; c Bradford School of Pharmacy, University of Bradford, West Yorkshire BD7 1DP, United Kingdom

**Keywords:** Trypanocidal agents, Antimalarial agents, 4-Aminoquinolines

## Abstract

A series of 19 huprines has been evaluated for their activity against cultured bloodstream forms of *Trypanosoma brucei* and *Plasmodium falciparum*. Moreover, cytotoxicity against rat myoblast L6 cells was assessed for selected huprines. All the tested huprines are moderately potent and selective trypanocidal agents, exhibiting IC_50_ values against *T. brucei* in the submicromolar to low micromolar range and selectivity indices for *T. brucei* over L6 cells of approximately 15, thus constituting interesting trypanocidal lead compounds. Two of these huprines were also found to be active against a chloroquine-resistant strain of *P. falciparum*, thus emerging as interesting trypanocidal–antiplasmodial dual acting compounds, but they exhibited little selectivity for *P. falciparum* over L6 cells.

## Graphical abstract

**Figure:**
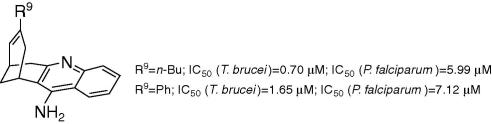

## 1 Introduction

Human African trypanosomiasis (HAT) and malaria are major protozoan parasite diseases in developing countries, with their main impact in the sub-Saharan Africa.^1^ Malaria is caused by *Plasmodium* species and infects several hundred million people annually, causing 1–3 million deaths. HAT, which results from infection with *Trypanosoma brucei gambiense* or *T. brucei rhodesiense*, currently kills 30,000 per year, although this can increase 10-fold during major epidemics.^2^, ^3^

**Figure 1:**
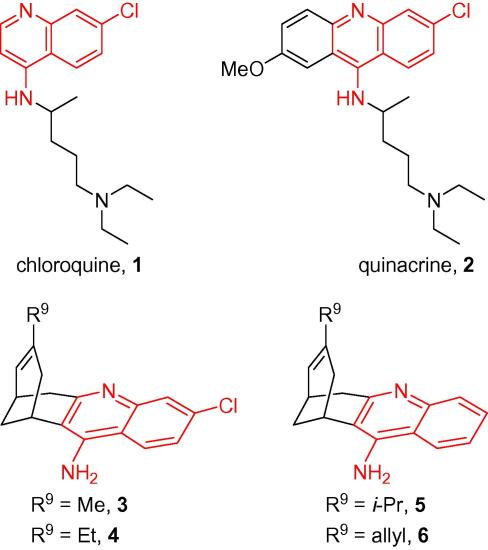
Structures of some antimalarial and trypanocidal 4-aminoquinoline-based drugs: chloroquine, quinacrine and some huprines.

Despite the mortality and morbidity caused by these diseases, conventional treatments are often inadequate.^4^, ^5^, ^6^ Of the drugs used against HAT, suramin and pentamidine are polar molecules which are highly charged at physiological pH, and cannot cross the blood–brain barrier (BBB). They are thus ineffective against late stage infection when, after an initial blood stream stage, parasites penetrate the central nervous system (CNS) giving rise to the classical symptoms of HAT. The arsenical melarsoprol is used to treat late stage disease, although it is extremely toxic, resulting in 5–10% mortality due to reactive encephalopathy.^2^, ^4^, ^7^ Cerebral malaria also causes severe disease episodes^8^ and brain penetration can also be an issue for antimalarial drugs. However, the main limitation in this case is the emergence of resistance, notably to chloroquine (**1**, Fig. 1), which was the mainstay of antimalarial chemotherapy for many decades.^8^, ^9^, ^10^, ^11^, ^12^

Given the problems with current treatments for HAT and malaria, there is a desperate need for new and better drugs.^5^, ^9^, ^11^, ^13^, ^14^ With malaria, alternative quinoline-based drugs bearing the 4-amino-7-chloroquinoline core of chloroquine are being assessed as a means of overcoming parasite resistance to the parent compound.^9^, ^15^, ^16^, ^17^, ^18^, ^19^ Interestingly, several 4-amino-7-chloroquinolines,^10^, ^20^ as well as closely related 9-amino-6-chloroacridines such as quinacrine (**2**, Fig. 1) and some derivatives thereof^7^, ^21^, ^22^, ^23^, ^24^, ^25^ exhibit both antiplasmodial and trypanocidal activity in vitro. Therefore, not only could aminoquinoline-based drugs circumvent the problem of resistant *Plasmodium* strains, but their dual antiplasmodial–trypanocidal profile could result in cost-effective treatments. Indeed, development of single agents which can act against multiple disease-causing organisms in an analogous way to broad-spectrum antibiotics would be an economic drug discovery approach of particular relevance to developing countries, where malaria and trypanosomiasis are endemic.^22^

Huprines constitute a class of 4-aminoquinoline-based compounds which were developed a decade ago as inhibitors of the enzyme acetylcholinesterase and were postulated as a treatment for Alzheimer’s disease.^26^, ^27^, ^28^, ^29^, ^30^ They have proved to be effective in the inhibition of brain acetylcholinesterase in ex vivo studies,^28^ while the so-called huprine X (**4**, Fig. 1), has been shown to ameliorate memory and learning activities in middle aged mice,^31^ as well as Alzheimer’s disease related brain neuropathology in a transgenic mouse model.^32^ These results are clearly indicative that huprines can cross the BBB and exert their actions within the CNS. Moreover, in the above-mentioned behavioural studies, huprine X did not induce adverse effects.

The profile and chemical structure of huprines prompted us to test them as a new family of trypanocidal–antiplasmodial brain permeable 4-aminoquinoline-based compounds. We recently reported that huprines **3–6** exhibited IC_50_ values in the range ∼300 nM for in vitro inhibition of the culture forms of *T. brucei* (strain 427).^33^ In the light of this moderately potent activity, we have now assessed the trypanocidal and antiplasmodial activity of a larger series of huprines. Herein, we report the synthesis of four novel huprines which combine the structural features of the previously tested huprines **3**–**6** (isopropyl and allyl groups at position 9 and a halogen atom on the benzene ring), and the evaluation of the in vitro activity of these and fifteen previously prepared huprines against cultured bloodstream forms of *T. brucei* and *Plasmodium falciparum* (strain K1). Furthermore, in vitro cytotoxic activity against rat skeletal myoblast L6 cells was assessed for selected huprines.

## 2 Results and discussion

### 2.1 Chemistry

**Scheme 1:**
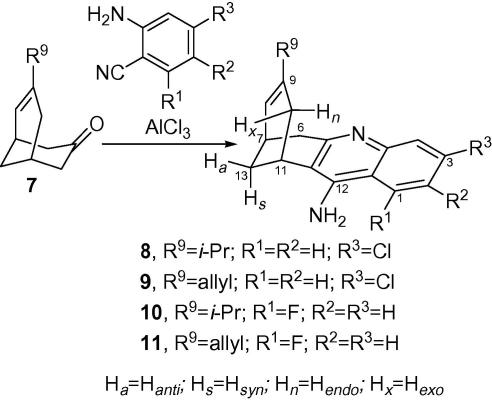
Synthesis of the novel huprines **8**–**11** and general structure of huprines **3**–**6** and **8**–**22**.

The novel huprines **8** and **9** were designed by combination of structural features of the recently tested trypanocidal huprines **3**–**6**, namely the chlorine atom at position 3 of huprines **3** and **4** and the isopropyl or allyl group at position 9 of huprines **5** and **6**. Introduction of a fluorine atom in a molecule often results in a series of added values, including enhanced binding interactions, metabolic stability or changes in physicochemical properties.^34^ In particular, the incorporation of a fluorine atom on the benzene ring of huprines will increase lipophilicity and decrease the basicity and hence the percent of protonation. This is likely to lead to better CNS penetration. Thus, the two novel huprines **10** and **11** were designed by combination of the isopropyl or allyl group at position 9 of huprines **5** and **6** with a fluorine atom at position 1 (Scheme 1).

For the synthesis of the novel huprines, the enones **7** (R^9^ = isopropyl or allyl) were prepared as previously described, that is, by nucleophilic addition of isopropylcerium chloride or allylmagnesium bromide to bicyclo[3.3.1]nonane-3,7-dione, followed by mesylation of the resulting 3-isopropyl- or 3-allyl-2-oxa-1-adamantanol and silica gel-promoted fragmentation.^27^ Friedländer condensation of these enones with commercially available 2-amino-4-chlorobenzonitrile or with 2-amino-6-fluorobenzonitrile in the presence of AlCl_3_ as Lewis acid catalyst in refluxing 1,2-dichloroethane afforded moderate yields of the novel huprines **8**–**11**, after column chromatography purification of the resulting crude product through silica gel (Scheme 1).

For antiparasitic evaluation, the novel huprines were converted into the corresponding hydrochlorides, which were fully characterized through their IR, ^1^H and ^13^C NMR spectra and elemental analysis.

Huprines **3**–**6** and **12**–**22** were prepared as previously described.^26^, ^27^, ^28^, ^29^

The ease and cost of the synthesis of antiparasitic drugs represent a major issue in developing countries. These huprines have been prepared by the above-mentioned four-step sequence from the readily available bicyclo[3.3.1]nonane-3,7-dione, which involves two rather expensive silica gel column chromatography purifications. Very interestingly, a three-step synthetic sequence for huprine derivatives has been reported, which avoids isolation of the intermediate enones **7**,^35^ and a recrystallization procedure has been set up for the purification of the final huprine derivatives, thus shortening the synthetic procedure, increasing its overall yield, and avoiding the two chromatographic purifications. The improved procedure would allow an easier, more efficient, and cheaper synthesis of our huprine derivatives, which is very convenient if they are intended to be used as antiparasitic drugs.

### 2.2 Trypanocidal activity and cytotoxicity

The in vitro activities of huprines **3**–**6** and **8**–**22** against cultured bloodstream forms *T. brucei*, as well as the toxicity of the most potent trypanocidal huprines to mammalian L6 cells, are shown in Table 1.

**Table 1:** Trypanocidal and cytotoxic activities of huprines^a^

Compd	R^1^	R^2^	R^3^	R^9^	*T. brucei* IC_50_ (μM)	*T. brucei* IC_90_ (μM)	L6 cells IC_50_ (μM)	SI^b^
**3**	H	H	Cl	Me	0.61 ± 0.03	2.94 ± 0.20	7.80 ± 0.47	12.8
**4**	H	H	Cl	Et	0.84 ± 0.06	1.76 ± 0.20	12.5 ± 0.40	14.9
**5**	H	H	H	*i*-Pr	2.06 ± 0.44	4.76 ± 0.82	nd^c^	
**6**	H	H	H	Allyl	4.08 ± 0.45	8.52 ± 0.19	nd	
**8**	H	H	Cl	*i*-Pr	2.45 ± 0.37	4.34 ± 0.03	nd	
**9**	H	H	Cl	Allyl	0.76 ± 0.07	2.87 ± 0.18	12.4 ± 0.40	16.3
**10**	F	H	H	*i*-Pr	2.60 ± 0.55	7.75 ± 0.23	nd	
**11**	F	H	H	Allyl	3.62 ± 0.54	8.73 ± 0.69	nd	
**12**	H	H	H	Et	1.29 ± 0.16	4.53 ± 0.16	15.6 ± 1.60	12.1
**13**	H	H	H	*n*-Bu	0.70 ± 0.01	0.97 ± 0.06	6.75 ± 0.49	9.6
**14**	H	H	H	*t*-Bu	0.88 ± 0.26	3.05 ± 0.03	11.6 ± 0.50	13.2
**15**	H	H	H	Ph	1.65 ± 0.27	4.70 ± 0.27	3.51 ± 0.14	2.1
**16**	F	H	H	Me	3.71 ± 0.56	15.8 ± 2.40	nd	
**17**	H	H	Me	Me	1.04 ± 0.05	3.31 ± 0.59	20.0 ± 0.50	19.2
**18**	Cl	H	H	Et	1.36 ± 0.19	3.11 ± 0.29	21.3 ± 1.30	15.7
**19**	F	H	H	Et	5.96 ± 0.58	9.50 ± 0.37	nd	
**20**	Me	H	H	Et	5.28 ± 1.11	8.86 ± 0.15	nd	
**21**	H	H	F	Et	4.16 ± 0.29	7.09 ± 0.29	nd	
**22**	H	Cl	H	Et	2.15 ± 0.27	5.02 ± 0.39	nd	

a Huprines were tested for in vitro activity against bloodstream form *T. brucei* (pH 7.4) and rat myoblast L6 cells and the concentrations that inhibited growth by 50% (IC_50_) and 90% (IC_90_, for trypanocidal activity) were calculated. Data are the mean of triplicate experiments ± SEM.

b SI: selectivity index is the ratio of cytotoxic to trypanocidal IC_50_ values.

c Not determined.

All of the compounds displayed trypanocidal potency, with IC_50_ values within the range 0.6–6 μM. Given the narrow range of potencies of the different huprines, no strong structure–activity relationship trends can be found.

The presence of a chlorine atom at position 3 seems to promote greater trypanocidal activity. 3-Chlorosubstituted huprines **3** and **4** displayed a similar potency against the cultured bloodstream forms *T. brucei* used in this assay to that found against the strain 427 of this parasite, with IC_50_ values in the submicromolar range.^33^ 9-Isopropyl- and 9-allyl-huprines **5** and **6** were found to be one order of magnitude less potent than previously described against the strain 427. Introduction of a chlorine atom at position 3 of huprines **5** and **6** led to the expected increased potency in the latter case, the novel 3-chloro-substituted huprine **9** being fivefold more potent than **6**, while huprine **8** was equipotent to the parent **5**.

Introduction of a chlorine atom at other positions of the benzene ring or introduction of other substituents onto the benzene ring, particularly a fluorine atom, was in general detrimental for the trypanocidal activity. Thus, the novel 1-fluoro-substituted huprines **10** and **11** were equipotent with the parent huprines **5** and **6**, while 1-fluoro- and 3-fluoro-substituted huprines **19** and **21** were 5- and 3-fold less potent than their parent huprine **12**, unsubstituted at the benzene ring. Also, when the activities of huprines with fluorine incorporations were compared with those in which chlorine atoms had been added (cf. compound **4** with **21**, and **18** with **19**), it was apparent that fluorine incorporation is associated with decreased activity. Therefore, although incorporation of fluorine atoms into the benzene ring may act to increase lipophilicity of the huprines, and potentially their CNS penetration, this is not reflected in an increased potency against trypanosomes in vitro.

Regarding the substitution at position 9, replacement of the isopropyl or allyl groups of huprines **5** and **6**, with either ethyl (**12**), butyl (**13**), *tert*-butyl (**14**) or phenyl (**15**) side chains, led in each case to a slight increase in trypanocidal activity. The most potent huprines unsubstituted at the benzene ring turned out to be those bearing a bulky butyl or *tert*-butyl group, which exhibited IC_50_ values in the submicromolar range.

The presence of a chlorine atom at position 3 is known to lead to an increased acetylcholinesterase inhibitory activity, while the contrary effect results from introduction of bulky substituents at position 9.^27^, ^28^ Indeed, 3-chloro-substituted huprines **3** and **4** are the most potent acetylcholinesterase inhibitors within the whole huprine family, while 9-butyl- and 9-*tert*-butyl-substituted huprines **13** and **14** are the least potent acetylcholinesterase inhibitors of the tested huprine series. Thus, the latter huprines seem to be more adequate as trypanocidal agents since their use should be associated with a lower incidence of cholinergically-mediated side effects.

Cytotoxicity for the most potent trypanocidal huprines against mammalian cells was evaluated in vitro, using cultured rat skeletal myoblast L6 cells. Even though the tested compounds displayed a modest toxicity toward the mammalian L6 cells with IC_50_ values ranging from 3.51 to 21.3 μM (Table 1), with the sole exception of compound **15** the tested huprines still possess selectivity indices for *T. brucei* over L6 myoblasts (SI = IC_50_ against L6 cells/IC_50_ against *T. brucei*) between 10 and 20, rendering them interesting trypanocidal lead compounds.

### 2.3 Antiplasmodial activity

The in vitro activities of huprines **3**–**6** and **8**–**22** against a chloroquine-resistant strain of *P. falciparum* (strain K1) were determined.

Only two of the nineteen huprine analogues, namely **13** and **15**, were found to have antiplasmodial activities (IC_50_) of less than 10 μM; their IC_50_ ± SD (*n* = 3) values, were 5.99 ± 1.64 and 7.12 ± 1.73 μM, respectively, as compared to 0.52 ± 0.11 μM for the positive control, chloroquine diphosphate. Although these activities are modest compared with that of chloroquine, it is noteworthy that both of these compounds, especially **13**, exhibited potent activities against trypanosomes.

In this study a chloroquine-resistant strain of *P. falciparum* was used but it would be of interest to evaluate **13** and **15** against a chloroquine-sensitive strain of *P. falciparum* to determine whether or not there is cross-resistance with chloroquine.

Huprine **13**, which displays a SI = 9.6 for *T. brucei* over L6 cells, is less selective toward *P. falciparum* (SI = 1.12), whereas huprine **15**, which exhibits a poor SI for *T. brucei* over L6 cells (SI = 2.1), is more toxic to L6 cells than to *P. falciparum* (SI = 0.49).

Thus, in spite of the interesting dual trypanocidal–antiplasmodial profile of huprines **13** and **15**, their therapeutic potential is limited by low selectivity for *P. falciparum* over L6 cells. The data also suggest that the antitrypanosomal and antiplasmodial activities of these compounds do not parallel each other, suggesting that their mode(s) of action against the two parasite species may be different.

## 3 Conclusions

Huprines have been found to display a moderately potent activity against cultured bloodstream forms *T. brucei*, particularly those bearing a chlorine atom at position 3 or a bulky butyl or *tert*-butyl substituent at position 9. Of these substitution patterns, the latter seems to be potentially more beneficial, taking into account that it leads to a decreased acetylcholinesterase inhibitory activity, and hence, it is likely to lead to a lower incidence of cholinergic side effects. Contrary to our expectations, huprines are in general not active against *P. falciparum*, despite bearing the 4-aminoquinoline or even the 4-amino-7-chloroquinoline core of the antimalarial drug chloroquine. Only the 9-butyl- and 9-phenyl-substituted huprines **13** and **15** were found to be active against the chloroquine-resistant strain K1 of *P. falciparum*. The most potent trypanocidal huprines display selectivity indices for *T. brucei* over rat skeletal myoblast L6 cells of approximately 15, thus constituting interesting trypanocidal lead compounds. However, their less favorable selectivity for *P. falciparum* over L6 cells, currently limits their potential as antiplasmodial agents, and hence as dual acting trypanocidal–antiplasmodial agents. Downstream development of huprines as antiparasitic compounds would be greatly enhanced by knowledge of their mechanism(s) of action and identification of their targets. In trypanosomes and *Plasmodium*, these are currently unknown, and on the basis of our data, may even be distinct. This information could be used to guide the modifications within this structural family that are needed to improve the antiplasmodial action and/or reduce cytotoxicity so that a convenient therapeutic window can also be achieved.

## 4 Experimental

### 4.1 Chemistry

#### 4.1.1 General

Melting points were determined in open capillary tubes with a MFB 595010 M Gallenkamp melting point apparatus. 500 MHz ^1^H NMR spectra and 75.4 MHz ^13^C NMR spectra were recorded in CD_3_OD on Varian Inova 500 or Brucker DMX500 and Varian Gemini 300 spectrometers, respectively. The chemical shifts are reported in ppm (*δ* scale) relative to internal TMS, and coupling constants are reported in Hertz (Hz). Assignments given for the NMR spectra of the new compounds have been carried out by comparison with the NMR data of tacrine (9-amino-1,2,3,4-tetrahydroacridine) and 6-chlorotacrine, as model compounds, which in turn, were assigned on the basis of DEPT, COSY ^1^H/^1^H (standard procedures), and COSY ^1^H/^13^C (gHMQC, gHSQC or gHMBC sequences) experiments. The *syn* (*anti*) notation of the protons at position 13 of huprines means that the corresponding proton at position 13 is on the same (different) side of the quinoline moiety with respect to the cyclohexene ring. IR spectra were run on a FT/IR Perkin–Elmer model 1600 spectrophotometer. Absorption values are expressed as wave-numbers (cm^−1^); only significant absorption bands are given. Column chromatography was performed on Silica Gel 60 AC.C (35–70 mesh, SDS, ref 2000027). Thin-layer chromatography (TLC) was performed with aluminum-backed sheets with Silica Gel 60 F254 (Merck, ref 1.05554), and spots were visualized with UV light and 1% aqueous solution of KMnO_4_. Analytical grade solvents were used for crystallization, while pure for synthesis solvents were used in the reactions, extractions and column chromatography. For characterization purposes, the new huprines **8**–**11** were transformed into the corresponding hydrochlorides and recrystallized. Worthy of note, as previously reported for some structurally related compounds,^36^ the new huprines have the ability to retain molecules of water and of solvents of crystallization, which cannot be removed after drying the analytical samples at 80 °C/1 Torr for 2 days. Thus, the elemental analyses of these compounds showed the presence of variable amounts of water and in some cases of EtOAc, used in the crystallization. NMR spectra of all of the new compounds were performed at the Serveis Cientificotècnics of the University of Barcelona, while elemental analyses were carried out at the Mycroanalysis Service of the IIQAB (CSIC, Barcelona, Spain), respectively.

#### 4.1.2 12-Amino-3-chloro-6,7,10,11-tetrahydro-9-isopropyl-7,11-methanocycloocta[*b*]quinoline hydrochloride, **8**·HCl

To a suspension of anhydrous AlCl_3_ (570 mg, 4.27 mmol) and freshly sublimed 2-amino-4-chlorobenzonitrile (618 mg, 4.05 mmol) in 1,2-dichloroethane (6 mL) was added dropwise a solution of enone **7** (R^9^ = *i*-Pr) (440 mg, 2.50 mmol) in 1,2-dichloroethane (27 mL). The reaction mixture was stirred under reflux for 14 h, allowed to cool to room temperature, diluted with water (35 mL) and THF (35 mL), made basic by addition of 5 N NaOH (20 mL), and stirred at room temperature for 30 min. The organic solvents were removed under reduced pressure, and the residue was filtered. The brown solid residue (698 mg) was subjected to column chromatography [silica gel (50 g), CH_2_Cl_2_/MeOH/25% aqueous NH_4_OH mixtures as eluent]. On elution with CH_2_Cl_2_/MeOH/25% aqueous NH_4_OH 98:2:0.5, huprine **8** (335 mg, 48% yield) was obtained as a beige solid.

A solution of huprine **8** (335 mg, 1.07 mmol) in CH_2_Cl_2_ (15 mL) was filtered through a polytetrafluoroethylene (PTFE) 0.45 μm filter, treated with 1.81 N methanolic solution of HCl (1.24 mL, 2.24 mmol), and the resulting solution was evaporated under reduced pressure. After recrystallization of the resulting solid residue from AcOEt/MeOH 8:1 (9 mL), **8**·HCl (274 mg) was obtained as a beige solid, mp 218–219 °C (AcOEt/MeOH 8:1). IR (KBr) *ν* 3500–2500 (max at 3476, 3322, 3172, 2956, 2926, 2892, 2821, C–H, N–H, and N^+^–H st), 1644, 1609, 1576, and 1557 (ar–C–C and ar–C–N st) cm^−1^; ^1^H NMR (500 MHz, CD_3_OD) *δ* 0.80 (d, *J* = 7.0 Hz, 3H) and 0.84 (d, *J* = 7.0 Hz, 3H) [9-CH(C*H*_3_)_2_], 1.92 (dm, *J* = 12.0 Hz, 1H, 13-H*_syn_*), 1.97–2.06 [complex signal, 2H, 13-H*_anti_* and 9-C*H*(CH_3_)_2_], 2.09 (d, *J* = 17.0 Hz, 1H, 10-H*_endo_*), 2.45 (dd, *J* = 17.0 Hz, *J*′ = 5.0 Hz, 1H, 10-H*_exo_*), 2.70 (m, 1H, 7-H), 2.82 (ddd, *J* = 17.5 Hz, *J*′ = *J*″ = 2.0 Hz, 1H, 6-H*_endo_*), 3.05 (dd, *J* = 17.5 Hz, *J*′ = 5.5 Hz, 1H, 6-H*_exo_*), 3.34 (m, 1H, 11-H), 4.86 (s, NH_2_ and NH^+^), 5.52 (dm, *J* = 5.0 Hz, 1H, 8-H), 7.30 (dd, *J* = 8.5 Hz, *J*′ = 2.0 Hz, 1H, 2-H), 7.66 (d, *J* = 2.0 Hz, 1H, 4-H), 8.06 (d, *J* = 8.5 Hz, 1H, 1-H); ^13^C NMR (75.4 MHz, CD_3_OD) *δ* 21.4 (CH_3_) and 21.9 (CH_3_) [9-CH(*C*H_3_)_2_], 28.2 (CH, C11), 29.2 (CH, C7), 30.4 (CH_2_, C13), 32.3 (CH_2_, C10), 35.9 [CH, 9-*C*H(CH_3_)_2_], 39.5 (CH_2_, C6), 115.5 (C) and 116.6 (C) (C11a and C12a), 122.9 (CH, C4), 124.6 (CH), 124.9 (CH), and 125.4 (CH) (C1, C2, and C8), 136.6 (C, C4a), 143.9 (C) and 145.9 (C) (C3 and C9), 151.4 (C) and 157.8 (C) (C5a and C12). Anal. Calcd for C_19_H_21_ClN_2_·HCl·1/3H_2_O (355.25): C, 64.24; H, 6.43; N, 7.89; Cl, 19.96. Found: C, 64.09; H, 6.45; N, 7.64; Cl, 19.70.

#### 4.1.3 9-Allyl-12-amino-3-chloro-6,7,10,11-tetrahydro-7,11-methanocycloocta[*b*]quinoline hydrochloride, **9**·HCl

It was prepared as described for **8**. Starting from freshly sublimed 2-amino-4-chlorobenzonitrile (618 mg, 4.05 mmol) in 1,2-dichloroethane (10 mL) and enone **7** (R^9^ = allyl) (475 mg, 2.70 mmol) in 1,2-dichloroethane (20 mL), and heating the reaction mixture under reflux for 18 h, a crude product (958 mg) was obtained and subjected to column chromatography [silica gel (75 g), CH_2_Cl_2_/MeOH/25% aqueous NH_4_OH mixtures as eluent]. On elution with CH_2_Cl_2_/MeOH/25% aqueous NH_4_OH 95:5:0.5, huprine **9** (163 mg, 19% yield) was obtained as a beige solid.

A solution of huprine **9** (163 mg, 0.50 mmol) in MeOH (5 mL) was treated with 1.81 N methanolic HCl (0.95 mL, 1.72 mmol) and the resulting solution was evaporated under reduced pressure. After recrystallization of the resulting solid residue from AcOEt/MeOH 4:1 (12 mL), **9**·HCl (171 mg) was obtained as a light brown solid, mp >300 °C (dec.) (AcOEt/MeOH 4:1). IR (KBr) *ν* 3500–2500 (max at 3338, 3174, 3080, 2926, 2903, 2821, 2681, C–H, N–H, and N^+^–H st), 1654, 1634, 1603, and 1586 (ar–C–C and ar–C–N st) cm^−1^; ^1^H NMR (500 MHz, CD_3_OD) *δ* 1.97 (dm, *J* = 12.5 Hz, 1H, 13-H*_syn_*), 2.04 (br d, *J* = 17.5 Hz, 1H, 10-H*_endo_*), 2.09 (dm, *J* = 12.5 Hz, 1H, 13-H*_anti_*), 2.50 (br dd, *J* = 17.5 Hz, *J*′ = 5.5 Hz, 1H, 10-H*_exo_*), 2.61 (d, *J* = 7.0 Hz, 2H, 9-C*H*_2_–CH=CH_2_), 2.83 (m, 1H, 7-H), 2.87 (dm, *J* = 18.0 Hz, 1H, 6-H*_endo_*), 3.22 (dd, *J* = 18.0 Hz, *J*′ = 5.5 Hz, 1H, 6-H*_exo_*), 3.39 (m, 1H, 11-H), 4.86 (s, NH_2_ and NH^+^), 4.84 (m, 1H) and 4.89 (m, 1H) (9-CH_2_–CH=C*H*_2_), 5.63 (dm, *J* ≈ 4.0 Hz, 1H, 8-H), superimposed in part 5.64 (ddt, *J* = 17.0 Hz, *J*′ = 10.0 Hz, *J*″ = 7.0 Hz, 1H, 9-CH_2_–C*H*=CH_2_), 7.58 (dd, *J* = 9.0 Hz, *J*′ = 2.0 Hz, 1H, 2-H), 7.75 (d, *J* ≈ 2.0 Hz, 1H, 4-H), 8.34 (d, *J* = 9.0 Hz, 1H, 1-H); ^13^C NMR (75.4 MHz, CD_3_OD) *δ* 27.6 (CH, C11), 28.2 (CH, C7), 29.3 (CH_2_, C13), 34.2 (CH_2_, C10), 36.1 (CH_2_, C6), 42.5 (CH_2_, 9-*C*H_2_–CH=CH_2_), 115.3 (C) and 115.4 (C) (C11a and C12a), 116.4 (CH_2_, 9-CH_2_–CH=*C*H_2_), 119.5 (CH, C4), 125.8 (CH, C8), 126.2 (CH, C1), 127.5 (CH, C2), 137.1 (CH, 9-CH_2_–*C*H=CH_2_), 137.2 (C, C9), 139.9 (C, C4a), 140.1 (C, C3), 153.2 (C) and 156.2 (C) (C5a and C12). Anal. Calcd for C_19_H_19_ClN_2_·HCl·H_2_O·0.2EtOAc (382.92): C, 62.11; H, 6.21; N, 7.32; Cl, 18.52. Found: C, 62.25; H, 5.92; N, 7.31; Cl, 18.18.

#### 4.1.4 12-Amino-1-fluoro-6,7,10,11-tetrahydro-9-isopropyl-7,11-methanocycloocta[*b*]quinoline hydrochloride, **10**·HCl

It was prepared as described for **8**. Starting from 2-amino-6-fluorobenzonitrile (318 mg, 2.31 mmol) in 1,2-dichloroethane (25 mL) and enone **7** (R^9^ = *i*-Pr) (272 mg, 1.53 mmol) in 1,2-dichloroethane (15 mL), a crude product (383 mg) was obtained and subjected to column chromatography [silica gel (19 g), hexane/AcOEt/25% aqueous NH_4_OH mixtures as eluent]. On elution with hexane/AcOEt/25% aqueous NH_4_OH 98:2:0.1, huprine **10** (220 mg, 42% yield) was obtained as a beige solid.

A solution of huprine **10** (220 mg, 0.74 mmol) in CH_2_Cl_2_ (15 mL) was filtered through a PTFE 0.45 μm filter, treated with 1.81 N methanolic solution of HCl (1.24 mL, 2.24 mmol), and the resulting solution was evaporated under reduced pressure. After recrystallization of the resulting solid residue from AcOEt/MeOH 30:1 (9.3 mL), **10**·HCl (138 mg) was obtained as a beige solid, mp >300 °C (dec.) (AcOEt/MeOH 30:1). IR (KBr) *ν* 3500–2500 (max at 3494, 3370, 3312, 3201, 3158, 3024, 2958, 2929, 2891, 2833, 2750, 2669, C–H, N–H, and N^+^–H st), 1640, 1595, and 1548 (ar–C–C and ar–C–N st) cm^−1^; ^1^H NMR (500 MHz, CD_3_OD) *δ* 0.87 (d, *J* = 7.0 Hz, 3H) and 0.89 (d, *J* = 7.0 Hz, 3 H) [9-CH(C*H*_3_)_2_], 1.97 (dm, *J* = 12.5 Hz, 1H, 13-H*_syn_*), 2.02–2.12 [complex signal, 2H, 13-H*_anti_* and 9-C*H*(CH_3_)_2_], superimposed in part 2.08 (br d, *J* = 17.5 Hz, 1H, 10-H*_endo_*), 2.52 (br dd, *J* = 17.5 Hz, *J*′ = 5.0 Hz, 1H, 10-H*_exo_*), 2.80 (m, 1H, 7-H), 2.86 (dm, *J* ≈ 18.0 Hz, 1H, 6-H*_endo_*), 3.21 (dd, *J* = 18.0 Hz, *J*′ = 5.5 Hz, 1H, 6-H*_exo_*), 3.41 (m, 1H, 11-H), 4.85 (s, NH_2_ and NH^+^), 5.58 (dm, *J* = 5.0 Hz, 1H, 8-H), 7.34 (ddd, *J* = 13.5 Hz, *J*′ = 8.0 Hz, *J*″ = 1.0 Hz, 1H, 2-H), 7.56 (br d, *J* = 8.5 Hz, 1H, 4-H), 7.82 (dddm, *J* ≈ *J*′ ≈ 8.5 Hz, *J*″ = 5.5 Hz, 1H, 3-H); ^13^C NMR (75.4 MHz, CD_3_OD) *δ* 21.4 (CH_3_) and 21.9 (CH_3_) [9-CH(*C*H_3_)_2_], 27.2 (CH, C11), 27.9 (CH, C7), 29.6 (CH_2_, C13), 31.7 (CH_2_, C10), 35.9 [CH, 9-*C*H(CH_3_)_2_], 36.1 (CH_2_, C6), 107.2 (C, d, *J* ≈ 11.5 Hz, C12a), 112.2 (CH, d, *J* = 23.3 Hz, C2), 115.7 (C, C11a), 116.3 (CH, C4), 122.2 (CH, C8), 134.9 (CH, d, *J* ≈ 11.5 Hz, C3), 140.6 (C, C4a), 144.5 (C, C9), 152.9 (C) and 155.3 (C) (C5a and C12), 161.1 (C, d, *J* = 253 Hz, C1). Anal. Calcd for C_19_H_21_FN_2_·HCl·3/4H_2_O (346.36): C, 65.89; H, 6.84; N, 8.09; Cl, 10.24. Found: C, 65.55; H, 6.62; N, 7.99; Cl, 10.21.

#### 4.1.5 9-Allyl-12-amino-1-fluoro-6,7,10,11-tetrahydro-7,11-methanocycloocta[*b*]quinoline hydrochloride, **11**·HCl

It was prepared as described for **8**. Starting from freshly sublimed 2-amino-6-fluorobenzonitrile (918 mg, 6.75 mmol) in 1,2-dichloroethane (8 mL) and enone **7** (R^9^ = allyl) (800 mg, 4.50 mmol) in 1,2-dichloroethane (40 mL), and heating the reaction mixture under reflux for 16 h, a crude product (1.75 g) was obtained and subjected to column chromatography [silica gel (62 g), CH_2_Cl_2_/MeOH/25% aqueous NH_4_OH mixtures as eluent]. On elution with CH_2_Cl_2_/MeOH/25% aqueous NH_4_OH 99:1:0.05, huprine **11** (762 mg, 58% yield) was obtained as a beige solid.

A solution of huprine **11** (762 mg, 2.59 mmol) in MeOH (6 mL) was treated with 1.81 N methanolic HCl (4.3 mL, 7.78 mmol) and the resulting solution was evaporated under reduced pressure. After recrystallization of the resulting solid residue from AcOEt/MeOH 15:2 (34 mL), **11**·HCl was obtained as a light brown solid (570 mg), mp >300 °C (dec.) (AcOEt/MeOH 15:2). IR (KBr) *ν* 3600–2400 (max at 3394, 3314, 3201, 3166, 3071, 3020, 2926, 2902, 2829, 2774, 2669, 2604, C–H, N–H, and N^+^–H st), 1640, 1593, and 1548 (ar–C–C and ar–C–N st) cm^−1^; ^1^H NMR (500 MHz, CD_3_OD) *δ* 1.98 (dm, *J* = 12.5 Hz, 1H, 13-H*_syn_*), 2.05 (br d, *J* ≈ 17.5 Hz, 1H, 10-H*_endo_*), 2.10 (dm, *J* = 12.5 Hz, 1H, 13-H*_anti_*), 2.51 (ddm, *J* = 17.5 Hz, *J*′ = 5.5 Hz, 1H, 10-H*_exo_*), 2.63 (d, *J* ≈ 7.0 Hz, 2H, 9-C*H*_2_–CH=CH_2_), 2.83 (m, 1H, 7-H), 2.88 (ddd, *J* = 18.0 Hz, *J*′ = *J*″ = 2.0 Hz, 1H, 6-H*_endo_*), 3.22 (dd, *J* = 18.0 Hz, *J*′ = 5.5 Hz, 1H, 6-H*_exo_*), 3.40 (m, 1H, 11-H), 4.86 (s, NH_2_ and NH^+^), 4.88–4.91 (complex signal, 2H, 9-CH_2_–CH=C*H*_2_), 5.63 (dm, *J* = 7.0 Hz, 1H, 8-H), superimposed in part 5.65 (ddt, *J* = 17.0 Hz, *J*′ = 10.0 Hz, *J*″ = 7.0 Hz, 1H, 9-CH_2_–C*H*=CH_2_), 7.34 (ddd, *J* = 14.0 Hz, *J*′ = 8.5 Hz, *J*″ = 1.0 Hz, 1H, 2-H), 7.57 (ddd, *J* = 8.5 Hz, *J*′ = *J*″ = 1.0 Hz, 1H, 4-H), 7.83 (ddd, *J* = *J*′ = 8.5 Hz, *J*″ = 6.0 Hz, 1H, 3-H); ^13^C NMR (75.4 MHz, CD_3_OD) *δ* 27.2 (CH, C11), 28.1 (CH, C7), 29.3 (CH_2_, C13), 34.0 (CH_2_, C10), 35.9 (CH_2_, C6), 42.5 (CH_2_, 9-*C*H_2_–CH=CH_2_), 107.4 (C, d, *J* = 12.1 Hz, C12a), 112.2 (CH, d, *J* = 23.0 Hz, C2), 115.8 (C, C11a), 116.3 (CH, C4), 116.4 (CH_2_, 9-CH_2_–CH=*C*H_2_), 125.7 (CH, C8), 135.0 (CH, d, *J* = 11.5 Hz, C3), 137.2 (CH, 9-CH_2_–*C*H=CH_2_), 137.3 (C, C9), 140.7 (C, C4a), 152.9 (C) and 155.4 (C) (C5a and C12), 161.2 (C, d, *J* = 253 Hz, C1). Anal. Calcd for C_19_H_19_FN_2_·HCl (330.83): C, 68.98; H, 6.09; N, 8.47; Cl, 10.72. Found: C, 68.65; H, 6.20; N, 8.35; Cl, 11.14.

### 4.2 *T. brucei* culturing and drug test

Bloodstream forms *T. brucei* (strain 427) were cultured at 37 °C in modified Iscove’s medium.^37^ Trypanocidal activity was assessed by growing parasites for 48 h in the presence of various drug concentrations and determining the levels which inhibited growth by 50% (IC_50_) and 90% (IC_90_). In the case of untreated cultures, cell densities increased from 0.25 × 10^5^ to 1 × 10^6^ over this period. Cell densities at each drug concentration were determined using a hemocytometer and drug sensitivity was expressed as a percentage of growth of control cells.

### 4.3 *P. falciparum* culturing and drug test

Malaria parasites were maintained in human A^+^ erythrocytes suspended in RPMI 1640 medium supplemented with A^+^ serum and d-glucose according to previously published methods.^38^, ^39^ Cultures containing predominantly early ring stages were used for testing. Compounds were dissolved in DMSO and further diluted with RPMI 1640 medium (the final DMSO concentration did not exceed 0.5% which did not affect parasite growth). Twofold serial dilutions were made in 96-well microtitre plates in duplicate and infected erythrocytes were added to give a final volume of 100 μL with haematocrit 2.5% and 1% parasitaemia. Chloroquine diphosphate was used as a positive control and uninfected and infected erythrocytes without compounds were included in each test. Plates were placed into a modular incubator gassed with nitrogen 93%, oxygen 3%, carbon dioxide 4% and incubated at 37 °C for 48 h. Parasite growth was assessed by measuring lactate dehydrogenase activity.^40^ The reagent used contained the following in each mL: acetylpyridine adenine dinucleotide (APAD), 0.74 mg; lithium lactate, 19.2 mg; diaphorase, 0.1 mg; triton X-100, 2 μL; and nitroblue tetrazolium, 1 mg. Reagent (50 μL) was added to each well and mixed, and plates were incubated for 10–15 min at 37 °C. Absorbances were read at 550 nm using a Dynatech Laboratories MRX microplate reader and % inhibition of growth was calculated by comparison with control values. IC_50_ values were determined using linear regression analysis (Microsoft Excel).

### 4.4 Cytotoxic activity against rat skeletal myoblast L6 cells

Cytotoxicity against mammalian cells was assessed using microtitre plates following a described procedure.^41^ Briefly, L6 cells (a rat skeletal muscle line) were seeded at 1 × 10^4^ ml^−1^ in 200 μL of growth medium containing different compound concentrations. The plates were incubated for 6 days at 37 °C and 20 μL Alamar Blue (Biosource UK Ltd) was then added to each well. After a further 8 h incubation, the fluorescence was determined using a Gemini fluorescent plate reader (Molecular Devices).
